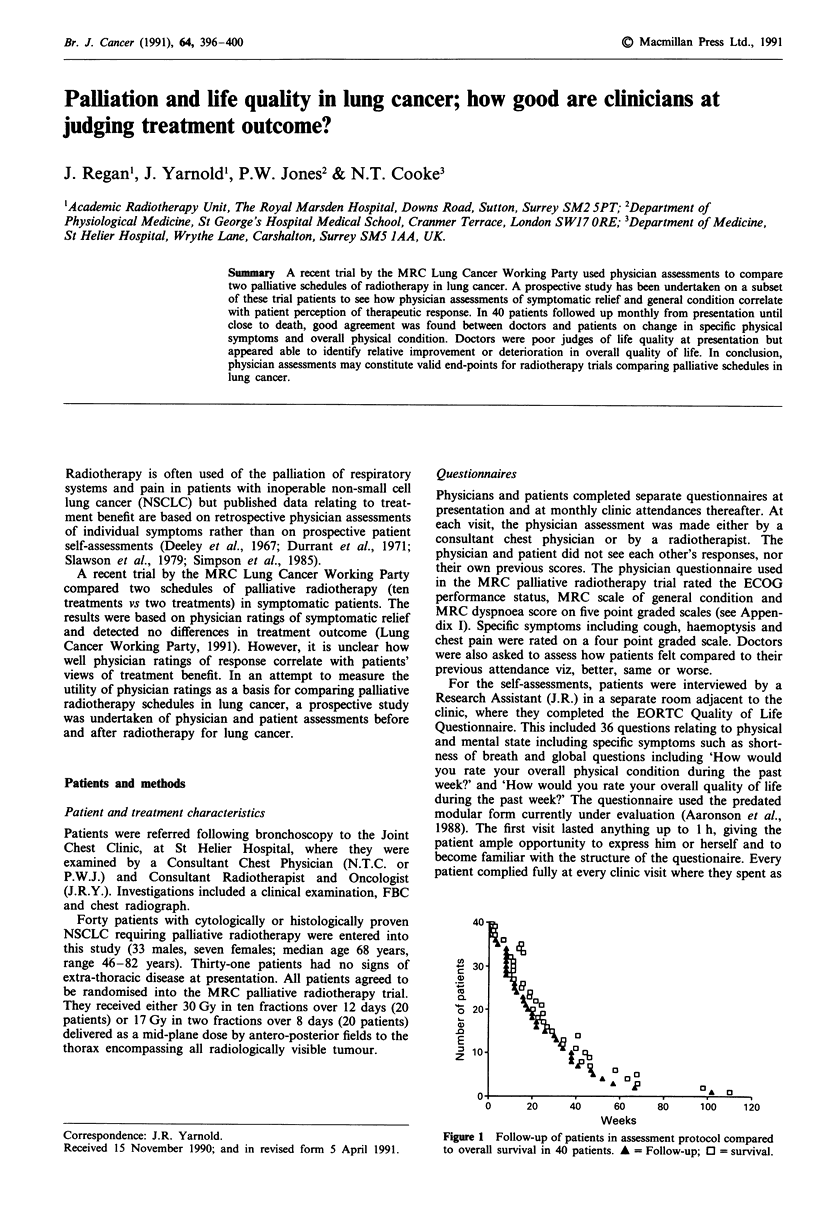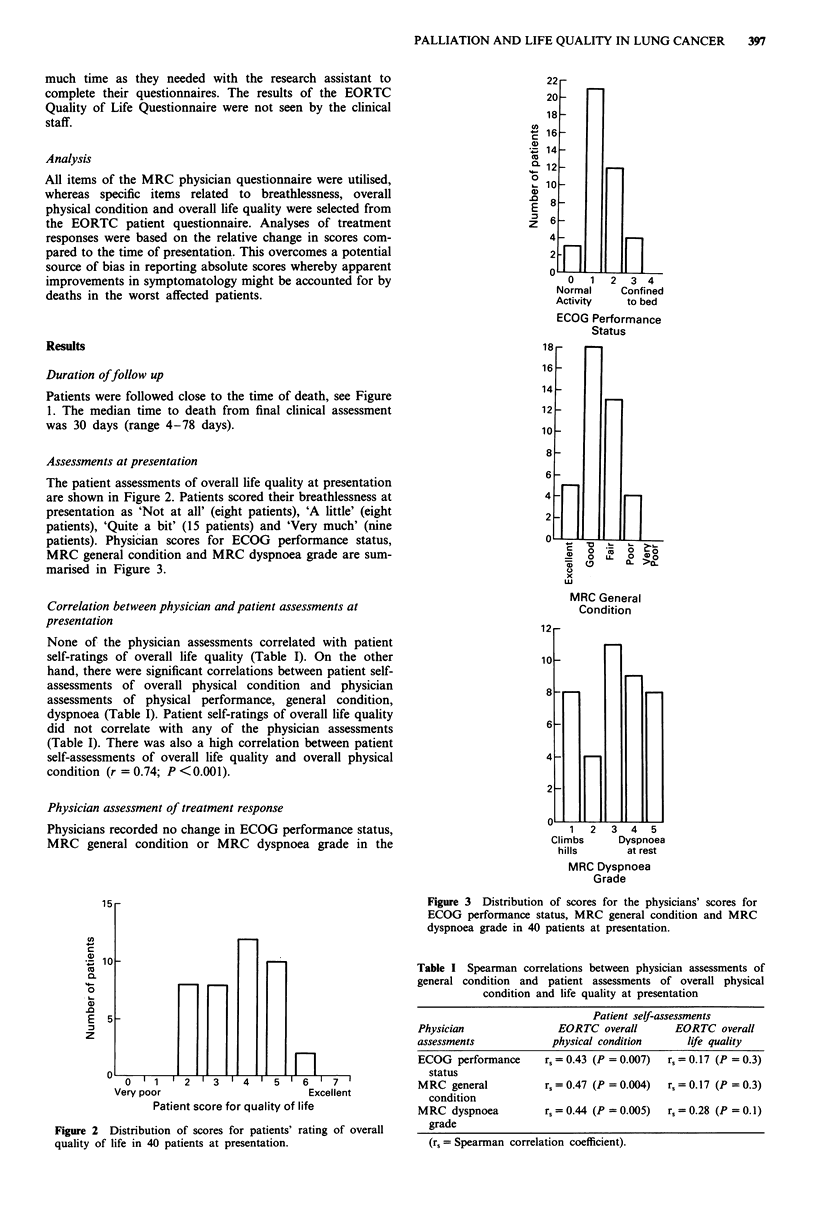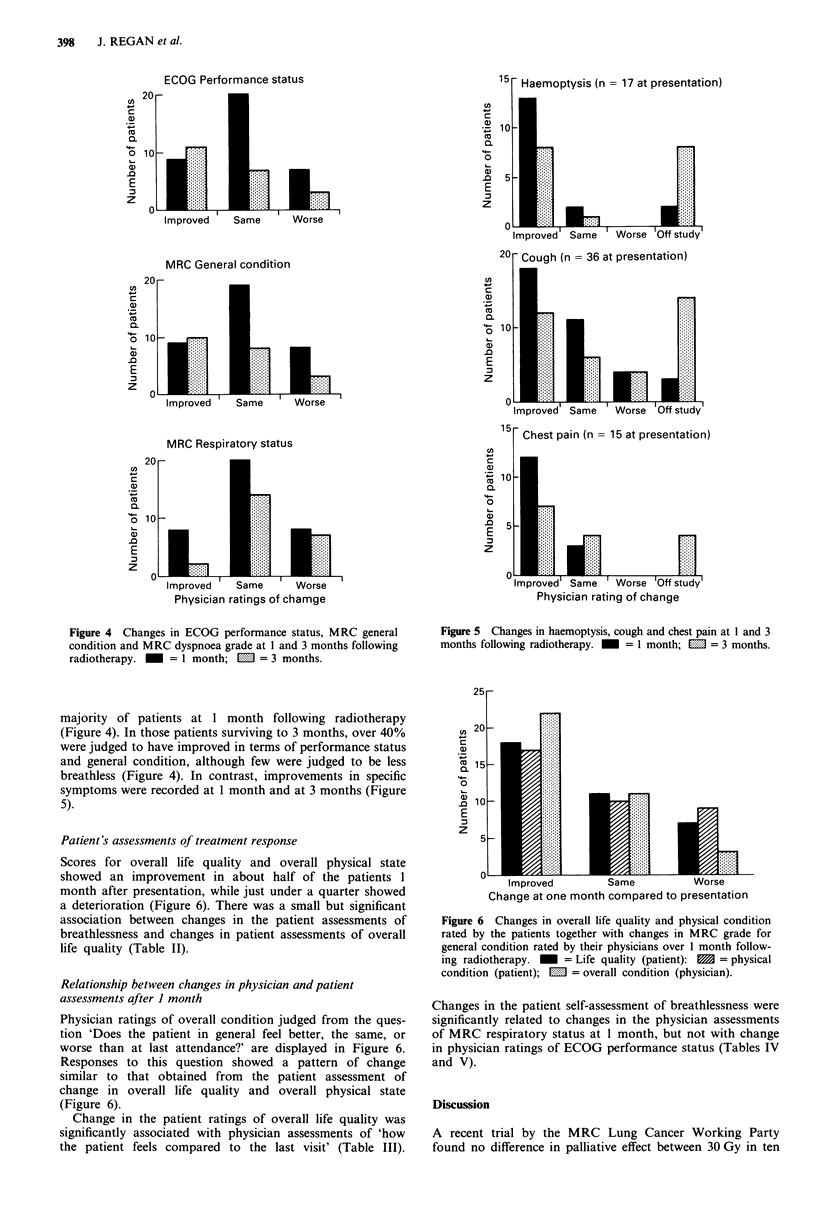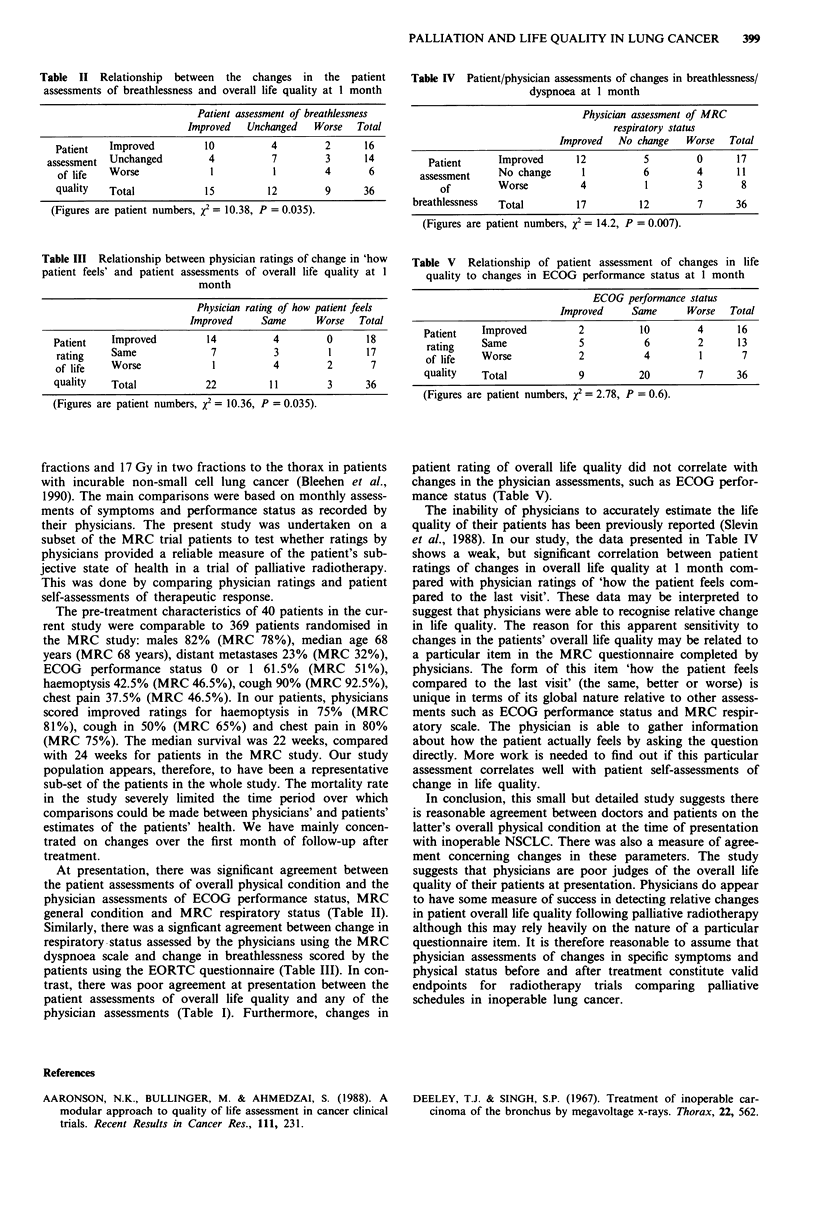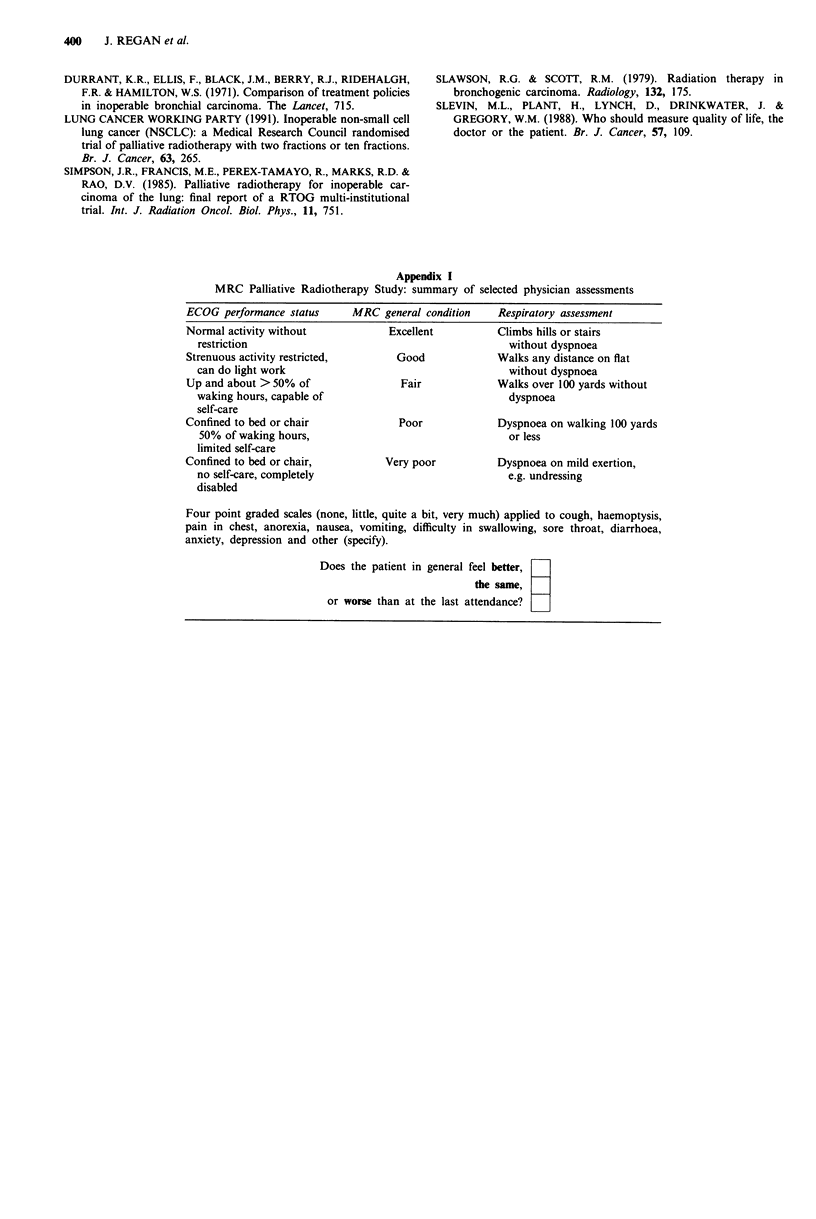# Palliation and life quality in lung cancer; how good are clinicians at judging treatment outcome?

**DOI:** 10.1038/bjc.1991.316

**Published:** 1991-08

**Authors:** J. Regan, J. Yarnold, P. W. Jones, N. T. Cooke

**Affiliations:** Academic Radiotherapy Unit, Royal Marsden Hospital, Sutton, Surrey, UK.

## Abstract

A recent trial by the MRC Lung Cancer Working Party used physician assessments to compare two palliative schedules of radiotherapy in lung cancer. A prospective study has been undertaken on a subset of these trial patients to see how physician assessments of symptomatic relief and general condition correlate with patient perception of therapeutic response. In 40 patients followed up monthly from presentation until close to death, good agreement was found between doctors and patients on change in specific physical symptoms and overall physical condition. Doctors were poor judges of life quality at presentation but appeared able to identify relative improvement or deterioration in overall quality of life. In conclusion, physician assessments may constitute valid end-points for radiotherapy trials comparing palliative schedules in lung cancer.


*Br. J. Cancer* (1991), **64**, 396–400 © Macmillan Press Ltd., 1991

### Palliation and life quality in lung cancer; how good are clinicians at judging treatment outcome?

J. Regan[1], J. Yarnold[1], P.W. Jones[2] & N.T. Cooke[3]

[1]*Academic Radiotherapy Unit, The Royal Marsden Hospital, Downs Road, Sutton, Surrey SM2 5PT;* [2]*Department of Physiological Medicine, St George's Hospital Medical School, Cranmer Terrace, London SW17 0RE;* [3]*Department of Medicine, St Helier Hospital, Wrythe Lane, Carshalton, Surrey SM5 1AA, UK.*

**Summary** A recent trial by the MRC Lung Cancer Working Party used physician assessments to compare two palliative schedules of radiotherapy in lung cancer. A prospective study has been undertaken on a subset of these trial patients to see how physician assessments of symptomatic relief and general condition correlate with patient perception of therapeutic response. In 40 patients followed up monthly from presentation until close to death, good agreement was found between doctors and patients on change in specific physical symptoms and overall physical condition. Doctors were poor judges of life quality at presentation but appeared able to identify relative improvement or deterioration in overall quality of life. In conclusion, physician assessments may constitute valid end-points for radiotherapy trials comparing palliative schedules in lung cancer.

Radiotherapy is often used of the palliation of respiratory systems and pain in patients with inoperable non-small cell lung cancer (NSCLC) but published data relating to treatment benefit are based on retrospective physician assessments of individual symptoms rather than on prospective patient self-assessments (Deeley *et al.*, 1967; Durrant *et al.*, 1971; Slawson *et al.*, 1979; Simpson *et al.*, 1985).

A recent trial by the MRC Lung Cancer Working Party compared two schedules of palliative radiotherapy (ten treatments *vs* two treatments) in symptomatic patients. The results were based on physician ratings of symptomatic relief and detected no differences in treatment outcome (Lung Cancer Working Party, 1991). However, it is unclear how well physician ratings of response correlate with patients' views of treatment benefit. In an attempt to measure the utility of physician ratings as a basis for comparing palliative radiotherapy schedules in lung cancer, a prospective study was undertaken of physician and patient assessments before and after radiotherapy for lung cancer.

### Patients and methods

### *Patient and treatment characteristics*

Patients were referred following bronchoscopy to the Joint Chest Clinic, at St Helier Hospital, where they were examined by a Consultant Chest Physician (N.T.C. or P.W.J.) and Consultant Radiotherapist and Oncologist (J.R.Y.). Investigations included a clinical examination, FBC and chest radiograph.

Forty patients with cytologically or histologically proven NSCLC requiring palliative radiotherapy were entered into this study (33 males, seven females; median age 68 years, range 46–82 years). Thirty-one patients had no signs of extra-thoracic disease at presentation. All patients agreed to be randomised into the MRC palliative radiotherapy trial. They received either 30 Gy in ten fractions over 12 days (20 patients) or 17 Gy in two fractions over 8 days (20 patients) delivered as a mid-plane dose by antero-posterior fields to the thorax encompassing all radiologically visible tumour.

Correspondence: J.R. Yarnold.
Received 15 November 1990; and in revised form 5 April 1991.

### *Questionnaires*

Physicians and patients completed separate questionnaires at presentation and at monthly clinic attendances thereafter. At each visit, the physician assessment was made either by a consultant chest physician or by a radiotherapist. The physician and patient did not see each other's responses, nor their own previous scores. The physician questionnaire used in the MRC palliative radiotherapy trial rated the ECOG performance status, MRC scale of general condition and MRC dyspnoea score on five point graded scales (see Appendix I). Specific symptoms including cough, haemoptysis and chest pain were rated on a four point graded scale. Doctors were also asked to assess how patients felt compared to their previous attendance viz, better, same or worse.

For the self-assessments, patients were interviewed by a Research Assistant (J.R.) in a separate room adjacent to the clinic, where they completed the EORTC Quality of Life Questionnaire. This included 36 questions relating to physical and mental state including specific symptoms such as shortness of breath and global questions including 'How would you rate your overall physical condition during the past week?' and 'How would you rate your overall quality of life during the past week?' The questionnaire used the predated modular form currently under evaluation (Aaronson *et al.*, 1988). The first visit lasted anything up to 1 h, giving the patient ample opportunity to express him or herself and to become familiar with the structure of the questionaire. Every patient complied fully at every clinic visit where they spent as

**Figure 1** Follow-up of patients in assessment protocol compared to overall survival in 40 patients. ▲ = Follow-up; □ = survival.

much time as they needed with the research assistant to complete their questionnaires. The results of the EORTC Quality of Life Questionnaire were not seen by the clinical staff.

*Analysis*

All items of the MRC physician questionnaire were utilised, whereas specific items related to breathlessness, overall physical condition and overall life quality were selected from the EORTC patient questionnaire. Analyses of treatment responses were based on the relative change in scores compared to the time of presentation. This overcomes a potential source of bias in reporting absolute scores whereby apparent improvements in symptomatology might be accounted for by deaths in the worst affected patients.

### Results

*Duration of follow up*

Patients were followed close to the time of death, see Figure 1. The median time to death from final clinical assessment was 30 days (range 4–78 days).

*Assessments at presentation*

The patient assessments of overall life quality at presentation are shown in Figure 2. Patients scored their breathlessness at presentation as 'Not at all' (eight patients), 'A little' (eight patients), 'Quite a bit' (15 patients) and 'Very much' (nine patients). Physician scores for ECOG performance status, MRC general condition and MRC dyspnoea grade are summarised in Figure 3.

*Correlation between physician and patient assessments at presentation*

None of the physician assessments correlated with patient self-ratings of overall life quality (Table I). On the other hand, there were significant correlations between patient self-assessments of overall physical condition and physician assessments of physical performance, general condition, dyspnoea (Table I). Patient self-ratings of overall life quality did not correlate with any of the physician assessments (Table I). There was also a high correlation between patient self-assessments of overall life quality and overall physical condition ($r = 0.74$; $P < 0.001$).

*Physician assessment of treatment response*

Physicians recorded no change in ECOG performance status, MRC general condition or MRC dyspnoea grade in the

**Figure 2** Distribution of scores for patients' rating of overall quality of life in 40 patients at presentation.

**Figure 3** Distribution of scores for the physicians' scores for ECOG performance status, MRC general condition and MRC dyspnoea grade in 40 patients at presentation.

**Table I** Spearman correlations between physician assessments of general condition and patient assessments of overall physical condition and life quality at presentation

| | *Patient self-assessments* | |
|---|---|---|
| *Physician assessments* | *EORTC overall physical condition* | *EORTC overall life quality* |
| ECOG performance status | $r_s = 0.43$ ($P = 0.007$) | $r_s = 0.17$ ($P = 0.3$) |
| MRC general condition | $r_s = 0.47$ ($P = 0.004$) | $r_s = 0.17$ ($P = 0.3$) |
| MRC dyspnoea grade | $r_s = 0.44$ ($P = 0.005$) | $r_s = 0.28$ ($P = 0.1$) |

($r_s$ = Spearman correlation coefficient).

Figure 4 Changes in ECOG performance status, MRC general condition and MRC dyspnoea grade at 1 and 3 months following radiotherapy. ■ = 1 month; ▒ = 3 months.

Figure 5 Changes in haemoptysis, cough and chest pain at 1 and 3 months following radiotherapy. ■ = 1 month; ▒ = 3 months.

majority of patients at 1 month following radiotherapy (Figure 4). In those patients surviving to 3 months, over 40% were judged to have improved in terms of performance status and general condition, although few were judged to be less breathless (Figure 4). In contrast, improvements in specific symptoms were recorded at 1 month and at 3 months (Figure 5).

*Patient's assessments of treatment response*

Scores for overall life quality and overall physical state showed an improvement in about half of the patients 1 month after presentation, while just under a quarter showed a deterioration (Figure 6). There was a small but significant association between changes in the patient assessments of breathlessness and changes in patient assessments of overall life quality (Table II).

*Relationship between changes in physician and patient assessments after 1 month*

Physician ratings of overall condition judged from the question 'Does the patient in general feel better, the same, or worse than at last attendance?' are displayed in Figure 6. Responses to this question showed a pattern of change similar to that obtained from the patient assessment of change in overall life quality and overall physical state (Figure 6).

Change in the patient ratings of overall life quality was significantly associated with physician assessments of 'how the patient feels compared to the last visit' (Table III).

Figure 6 Changes in overall life quality and physical condition rated by the patients together with changes in MRC grade for general condition rated by their physicians over 1 month following radiotherapy. ■ = Life quality (patient): ▨ = physical condition (patient); ▒ = overall condition (physician).

Changes in the patient self-assessment of breathlessness were significantly related to changes in the physician assessments of MRC respiratory status at 1 month, but not with change in physician ratings of ECOG performance status (Tables IV and V).

### Discussion

A recent trial by the MRC Lung Cancer Working Party found no difference in palliative effect between 30 Gy in ten

**Table II** Relationship between the changes in the patient assessments of breathlessness and overall life quality at 1 month

| | | *Patient assessment of breathlessness* | | | |
|---|---|---|---|---|---|
| | | *Improved* | *Unchanged* | *Worse* | *Total* |
| Patient assessment of life quality | Improved | 10 | 4 | 2 | 16 |
| | Unchanged | 4 | 7 | 3 | 14 |
| | Worse | 1 | 1 | 4 | 6 |
| | Total | 15 | 12 | 9 | 36 |

(Figures are patient numbers, $\chi^2 = 10.38$, $P = 0.035$).

**Table III** Relationship between physician ratings of change in 'how patient feels' and patient assessments of overall life quality at 1 month

| | | *Physician rating of how patient feels* | | | |
|---|---|---|---|---|---|
| | | *Improved* | *Same* | *Worse* | *Total* |
| Patient rating of life quality | Improved | 14 | 4 | 0 | 18 |
| | Same | 7 | 3 | 1 | 17 |
| | Worse | 1 | 4 | 2 | 7 |
| | Total | 22 | 11 | 3 | 36 |

(Figures are patient numbers, $\chi^2 = 10.36$, $P = 0.035$).

**Table IV** Patient/physician assessments of changes in breathlessness/dyspnoea at 1 month

| | | *Physician assessment of MRC respiratory status* | | | |
|---|---|---|---|---|---|
| | | *Improved* | *No change* | *Worse* | *Total* |
| Patient assessment of breathlessness | Improved | 12 | 5 | 0 | 17 |
| | No change | 1 | 6 | 4 | 11 |
| | Worse | 4 | 1 | 3 | 8 |
| | Total | 17 | 12 | 7 | 36 |

(Figures are patient numbers, $\chi^2 = 14.2$, $P = 0.007$).

**Table V** Relationship of patient assessment of changes in life quality to changes in ECOG performance status at 1 month

| | | *ECOG performance status* | | | |
|---|---|---|---|---|---|
| | | *Improved* | *Same* | *Worse* | *Total* |
| Patient rating of life quality | Improved | 2 | 10 | 4 | 16 |
| | Same | 5 | 6 | 2 | 13 |
| | Worse | 2 | 4 | 1 | 7 |
| | Total | 9 | 20 | 7 | 36 |

(Figures are patient numbers, $\chi^2 = 2.78$, $P = 0.6$).

fractions and 17 Gy in two fractions to the thorax in patients with incurable non-small cell lung cancer (Bleehen *et al.*, 1990). The main comparisons were based on monthly assessments of symptoms and performance status as recorded by their physicians. The present study was undertaken on a subset of the MRC trial patients to test whether ratings by physicians provided a reliable measure of the patient's subjective state of health in a trial of palliative radiotherapy. This was done by comparing physician ratings and patient self-assessments of therapeutic response.

The pre-treatment characteristics of 40 patients in the current study were comparable to 369 patients randomised in the MRC study: males 82% (MRC 78%), median age 68 years (MRC 68 years), distant metastases 23% (MRC 32%), ECOG performance status 0 or 1 61.5% (MRC 51%), haemoptysis 42.5% (MRC 46.5%), cough 90% (MRC 92.5%), chest pain 37.5% (MRC 46.5%). In our patients, physicians scored improved ratings for haemoptysis in 75% (MRC 81%), cough in 50% (MRC 65%) and chest pain in 80% (MRC 75%). The median survival was 22 weeks, compared with 24 weeks for patients in the MRC study. Our study population appears, therefore, to have been a representative sub-set of the patients in the whole study. The mortality rate in the study severely limited the time period over which comparisons could be made between physicians' and patients' estimates of the patients' health. We have mainly concentrated on changes over the first month of follow-up after treatment.

At presentation, there was significant agreement between the patient assessments of overall physical condition and the physician assessments of ECOG performance status, MRC general condition and MRC respiratory status (Table II). Similarly, there was a signficant agreement between change in respiratory status assessed by the physicians using the MRC dyspnoea scale and change in breathlessness scored by the patients using the EORTC questionnaire (Table III). In contrast, there was poor agreement at presentation between the patient assessments of overall life quality and any of the physician assessments (Table I). Furthermore, changes in patient rating of overall life quality did not correlate with changes in the physician assessments, such as ECOG performance status (Table V).

The inability of physicians to accurately estimate the life quality of their patients has been previously reported (Slevin *et al.*, 1988). In our study, the data presented in Table IV shows a weak, but significant correlation between patient ratings of changes in overall life quality at 1 month compared with physician ratings of 'how the patient feels compared to the last visit'. These data may be interpreted to suggest that physicians were able to recognise relative change in life quality. The reason for this apparent sensitivity to changes in the patients' overall life quality may be related to a particular item in the MRC questionnaire completed by physicians. The form of this item 'how the patient feels compared to the last visit' (the same, better or worse) is unique in terms of its global nature relative to other assessments such as ECOG performance status and MRC respiratory scale. The physician is able to gather information about how the patient actually feels by asking the question directly. More work is needed to find out if this particular assessment correlates well with patient self-assessments of change in life quality.

In conclusion, this small but detailed study suggests there is reasonable agreement between doctors and patients on the latter's overall physical condition at the time of presentation with inoperable NSCLC. There was also a measure of agreement concerning changes in these parameters. The study suggests that physicians are poor judges of the overall life quality of their patients at presentation. Physicians do appear to have some measure of success in detecting relative changes in patient overall life quality following palliative radiotherapy although this may rely heavily on the nature of a particular questionnaire item. It is therefore reasonable to assume that physician assessments of changes in specific symptoms and physical status before and after treatment constitute valid endpoints for radiotherapy trials comparing palliative schedules in inoperable lung cancer.

### References

AARONSON, N.K., BULLINGER, M. & AHMEDZAI, S. (1988). A modular approach to quality of life assessment in cancer clinical trials. *Recent Results in Cancer Res.*, **111**, 231.

DEELEY, T.J. & SINGH, S.P. (1967). Treatment of inoperable carcinoma of the bronchus by megavoltage x-rays. *Thorax*, **22**, 562.

DURRANT, K.R., ELLIS, F., BLACK, J.M., BERRY, R.J., RIDEHALGH, F.R. & HAMILTON, W.S. (1971). Comparison of treatment policies in inoperable bronchial carcinoma. The *Lancet*, 715.

LUNG CANCER WORKING PARTY (1991). Inoperable non-small cell lung cancer (NSCLC): a Medical Research Council randomised trial of palliative radiotherapy with two fractions or ten fractions. *Br. J. Cancer*, **63**, 265.

SIMPSON, J.R., FRANCIS, M.E., PEREX-TAMAYO, R., MARKS, R.D. & RAO, D.V. (1985). Palliative radiotherapy for inoperable carcinoma of the lung: final report of a RTOG multi-institutional trial. *Int. J. Radiation Oncol. Biol. Phys.*, **11**, 751.

SLAWSON, R.G. & SCOTT, R.M. (1979). Radiation therapy in bronchogenic carcinoma. *Radiology*, **132**, 175.

SLEVIN, M.L., PLANT, H., LYNCH, D., DRINKWATER, J. & GREGORY, W.M. (1988). Who should measure quality of life, the doctor or the patient. *Br. J. Cancer*, **57**, 109.

**Appendix I**

MRC Palliative Radiotherapy Study: summary of selected physician assessments

| *ECOG performance status* | *MRC general condition* | *Respiratory assessment* |
|---|---|---|
| Normal activity without restriction | Excellent | Climbs hills or stairs without dyspnoea |
| Strenuous activity restricted, can do light work | Good | Walks any distance on flat without dyspnoea |
| Up and about >50% of waking hours, capable of self-care | Fair | Walks over 100 yards without dyspnoea |
| Confined to bed or chair 50% of waking hours, limited self-care | Poor | Dyspnoea on walking 100 yards or less |
| Confined to bed or chair, no self-care, completely disabled | Very poor | Dyspnoea on mild exertion, e.g. undressing |

Four point graded scales (none, little, quite a bit, very much) applied to cough, haemoptysis, pain in chest, anorexia, nausea, vomiting, difficulty in swallowing, sore throat, diarrhoea, anxiety, depression and other (specify).

Does the patient in general feel **better**, ☐

**the same**, ☐

or **worse** than at the last attendance? ☐